# A Statistical Model of Fibre Distribution in a Steel Fibre Reinforced Concrete

**DOI:** 10.3390/ma14237297

**Published:** 2021-11-29

**Authors:** Janusz Kobaka

**Affiliations:** Faculty of Geoengineering, University of Warmia and Mazury in Olsztyn, 10-720 Olsztyn, Poland; janusz.kobaka@uwm.edu.pl

**Keywords:** SFRC, model, fibre, distribution, statistical probability

## Abstract

The aim of the research was to create a model of steel fibre distribution in a Steel Fibre Reinforced Concrete space using statistical probability means. The model was created in order to better understand the behaviour of the composite under operating conditions. Four statistical distributions (Beta, Kumaraswamy, Three Parameter Beta and Generalised Transmuted Kumaraswamy) were examined to find the distribution that best described fibre settling phenomenon caused by manufacturing process conditions. In the next stage the chosen statistical distribution was adapted to create the model of steel fibre distribution in a Steel Fibre Reinforced Concrete space. The model took into account technological conditions such as vibrating time and properties such as consistency of the tested concrete. The model showed a good agreement with the real fibre distribution.

## 1. Introduction

Steel Fibre Reinforced Concrete (SFRC) is a widely described composite construction material of constantly growing popularity due to some improved properties in comparison to ordinary concrete. The addition of steel fibres influences for instance the flexural strength of a concrete transforming it from brittle to quasi-plastic material [1]. It also significantly improves post-cracking properties under tension [2] and increases the strength in impact tests [3,4,5,6,7], affects well long-term performance when exposed to aggressive environment [8]. The addition of fibres in concrete develops the stress transfer mechanism by bridging cracks [9]. Due to good physical and mechanical parameters of SFRC it is applied for making various structural elements such as foundation slabs [10], bridge slabs [11], industrial floors [12], columns [13,14] or tunnel elements [15,16,17,18]. In SFRC production waste materials can also be applied [19] bearing in mind sustainable development of construction industry.

Steel fibres are generally assumed to be randomly distributed in concrete and SFRC is treated as an isotropic material [20,21,22,23]. In reality, the fibre distribution is a complex problem. Real fibre orientation in a composite space is unknown. Fibres are oriented at various angles and located in various distances between themselves and walls of a mould or formwork [24]. There are many factors that affect fibre distribution such as the length and the slenderness of fibres, properties of a concrete matrix (its consistency, aggregate characteristics), technological process of making concrete (mixing and vibrating time) [12]. Increasing the fibre content from 1% to 2% can increase the plastic viscosity of concrete, which might improve the fibre distribution [25]. The irregularity in fibre distribution significantly affects properties of SFRC [26]. The distribution of steel fibres has a key influence on basic mechanical properties of the composite, such as tensile strength, the modulus of elasticity, or impact strength [12]. During the production process of a concrete element steel fibres tend to settle down (because of the gravity) which change the mechanical characteristics of cast elements. Fibre distribution along depth mainly depends on rheological parameters of fresh mixture [27].

Description of the fibre distribution in the composite space is not a new issue. Various methods were used to describe this phenomenon such as geometric method [28,29] or probabilistic method [30]. When describing fibre distribution by statistical means, the probability of fibre occurrence in a given fragment of a composite space is considered. Taking into account the simplified model of fibre distribution when steel fibres are randomly distributed in the composite space, the continuous uniform distribution is the best solution to describe fibre distribution along the horizontal axes of cast element. When taking into account fibre distribution disturbance of regularity such as settling down of fibres along vertical axis, one should apply a statistical distribution that can be adjusted accordingly.

There are some probability functions which could describe fibre distribution. In previous study the author described fibre distribution using Kumaraswamy distribution [12]. In this study the author made an attempt to compare four probability models (Beta, Kumaraswamy, Three Parameter Beta and Generalised Transmuted Kumaraswamy) and answer the question which distribution will provide the best fit to fibre distribution disturbance. The irregularities in SFRC space caused by technological conditions during making the concrete and the properties of the concrete itself should be mirrored by the chosen probability distribution. The aim of this research study was comparison and adaptation of statistical distributions in order to describe fibre distribution in the SFRC space taking into account fibre settlement caused by technological conditions during making the concrete and the properties of the concrete itself. The anticipated result was the creation of a statistical model describing these phenomena.

## 2. Description of the Discussed Probability Distributions

The Beta probability distribution originates from 1676 when Isaac Newton wrote a letter to Henry Oldenbeg where he evaluated $\int_{0}^{x}\mathrm{ydz}$ as series [31]. Since then Beta family of distributions has been applied extensively in statistical theory and practice [32]. Probability density function (pdf) and cumulative distribution function (cdf) of Beta distribution are given by the following equations:

(1) $$f\left(x;\;a,\;b\right)=\frac{1}{B\left(a,b\right)}x^{a-1}{\left(1-x\right)}^{b-1},\;\;\;\;0\;<\;x\;<\;1\;$$

(2) $$F\left(x;\;a,\;b\right)=\frac{B\left(x;\;a,b\right)}{B\left(a,b\right)},\;\;\;\;\;\;\;\;0\;<\;x\;<\;1\;$$

Kumaraswamy distribution was proposed in 1980 by Poondi Kumaraswamy in order to describe hydrological random variables such as daily rainfall and daily stream flow [33,34]. The Kumaraswamy distribution is a very flexible model in which density function can be unimodal, increasing, decreasing, or constant depending on the values of its parameters [35]. The model shows well goodness-of-fit for many natural phenomena [36]. Pdf and cdf of Kumaraswamy distribution are given by the following equations:

(3) $$f\left(x;\;\alpha ,\;\beta \right)={\alpha \beta x}^{\alpha -1}{\left(1-x^{\alpha }\right)}^{\beta -1},\;\;\;0\;<\;x\;<\;1\;$$

(4) $$F\left(x;\;\alpha ,\;\beta \right)=1-{\left(1-x^{\alpha }\right)}^{\beta },\;\;\;\;0\;<\;x\;<\;1\;$$

Three Parameter Beta (TPB) distribution derived in 1984 by James B McDonald [37] is a generalised Beta distribution type [35]. Classical Beta distribution is a special case of the TPB distribution when (p, γ, δ) = (1, a, b). Kumaraswamy distribution is a special case of the TPB distribution when (p, γ, δ) = (α, 1, β) [35]. Pdf and cdf of the Three-Parameter Beta (TPB) distribution [35] are described by the following equations:

(5) $$f\left(x;\;p,\;\gamma ,\;\delta \right)=\frac{p}{B\left(\gamma ,\;\delta \right)}x^{\gamma p-1}{\left(1-x\right)}^{\delta -1},\;\;\;\;\;\;0\;<\;x\;<\;1\;$$

(6) $$F\left(x;\;p,\;\gamma ,\;\delta \right)=\frac{p}{B\left(\gamma ,\;\delta \right)}\int_{0}^{x}x^{\gamma p-1}{\left(1-x\right)}^{\delta -1}{\;\mathrm{dx}}^{\;},\;\;\;0\;<\;x\;<\;1\;$$

Generalised Transmuted Kumaraswamy (GT-Kw) distribution was proposed in 2019 by Aliyu Ishaq at al. [38] in order to generate a flexible distribution as an extension of the Kumaraswamy distribution based on Generalized Transmuted—G family pioneered by Nofal et al. [38,39]. Pdf and cdf of GTK are described as follows:

(7) $$f\left(x;\;\lambda ,\;a,\;b,\;c,\;d\;\right)={\mathrm{cdx}}^{c-1}{\left(1-x^{c}\right)}^{d}{)}^{a}[\left(1+\lambda \right)-\lambda (1-\left(1-x^{c}{)}^{d}{)}^{b}\right],\;a,b,c,d\;>\;0,\;|\lambda |\;<\;1\;$$

(8) $$\left(x;\;\lambda ,\;a,\;b,\;c,\;d\;\right)={\left(1-{\left(1-x^{c}\right)}^{d}\right)}^{a}[\left(1+\lambda \right)-\lambda (1-\left(1-x^{c}{)}^{d}{)}^{b}\right],\;a,b,c,d\;>\;0,\;|\lambda |\;<\;1$$

## 3. Materials, Methods, and Experiment Design

The experiment design assumed conducting the research on specimens that during casting were characterised by different consistency (according to Vebe method) and vibration time (see Table 1). The desired consistency was achieved by adding various amount of water (see Table 2).

The choice of hooked type of fibre was due to its popularity in the global civil and structural engineering market [1,42]. Twenty-seven cubic specimens with a side of 15 cm were formed.

In order to simulate fibre distribution in a given space of dimensions the same as the tested cubic specimens, a computer self-authored program named CHI-Curvefit 2.0 was applied. The program was created in 2021 and based on Visual Basic for Applications. The four statistical distributions described by the Equations (1)–(8) were used to find the best fit with tested distributions of fibres along the vertical axis The search for the best fit was based on chi-square test measuring goodness of fit. The purpose of the program was to check out what values of distribution parameters provide the best fit. The program also compared the calculation results with the critical chi-square value calculated for the given number of degrees of freedom and the significance level equal to 5%.

Another self-authored program Fiberdist 2.0 [12] written in Statistica Visual Basic was applied. The program generated within the cubic space Cartesian position coordinates of all the fibres. To achieve this purpose along the two horizontal axes a uniform probability distribution was used, along the vertical axis GT-Kw distribution was used with parameters corresponding to the vibration time and consistency. At the first stage the coordinates of a point understood as the end of the fibre were generated. Then the tilt angle of the fibre was generated using uniform distribution and the coordinates of the opposite end of the fibre. The coordinates were calculated taking into account the length of the fibre. If the coordinates were outside the cubic space the calculations of the angle were repeated. Thus the coordinates of such a number of fibres were generated as to correspond to the content of 1.5% of fibre volume fraction. After generating the coordinates of all the fibres within the model of cubic space, the space was “cut” mathematically in half. The points of “piercing” the cross-section plane by the calculated fibres were computed and the image of the cross-section plane was generated.

## 4. Results

**Figure 1:**
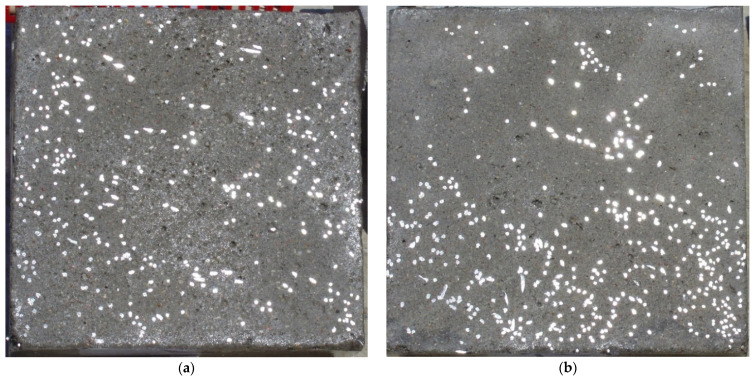
Cross-sections of tested SFRC cubic specimens differentiated by vibration time and consistency: (**a**) *t_vib_* = 20 s, *t_Veb e_* = 7 s, (**b**) *t_vib_* = 240 s, *t_Vebe_* = 2 s.

After 28 days of curing, specimens were cut in half along the vertical axis (see Figure 1). Varied sedimentation of steel fibres in the specimens depending on vibration time and consistency was noticed. By comparing the cross-section of specimen characterized by medium workability and short vibration time (see Figure 1a) with the cross-section of specimen of fluid mix and long vibration time (Figure 1b) one can see the tendency of settling down of steel fibres.

**Figure 2:**
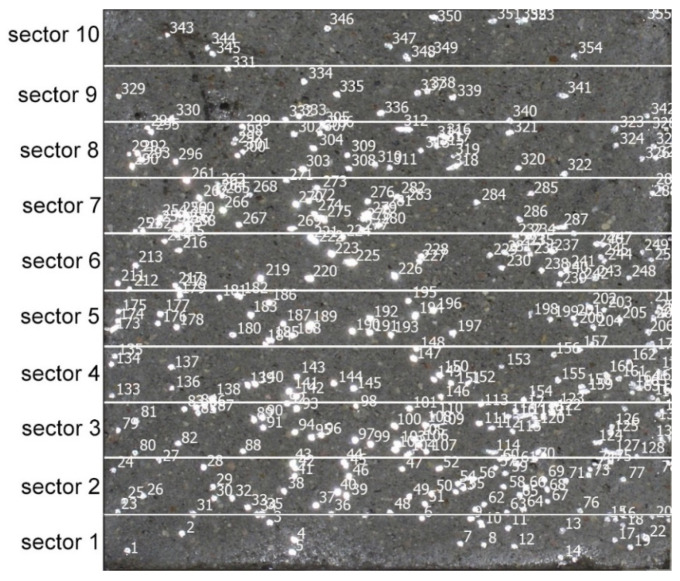
Exemplary cross-section of a SFRC specimen divided into 10 sectors with fibres counted.

Each specimen’s cross-section was divided into 10 sectors. Overall number of fibres and numbers of fibres in each sector was counted (see Figure 2). Mean value of fibre amount for each sector was counted for three specimens of each vibrating time and consistency (see Table 1).

The next step was to determine parameters for four types of statistical distributions: Beta, Kumaraswamy TPB, and GT-Kw. Computer program CHI-Curvefit was applied searching for parameters which will ensure the best fit of the statistical distribution to the real fibre distribution along the *Z* axis. The parameters were calculated separately for different vibrating time and consistency of concrete (see Table 3).

All calculation results performed by computer program *CHI*-Curvefit for four statistical distributions were lower than critical chi-square value equal to 14.067 for the assumed probability and degrees of freedom which fulfilled statistical requirements for the chi-square test (see Table 4). The lower value of chi-square test the better curve fitting. The best fitting results were achieved for GT-Kw distribution. The worst result of fitting was recorded for the two parameter Kumaraswamy distribution though still fulfilling the statistical requirements. Multi-parameter distributions (TPB and GT-Kw) are more complex in calculations and require more time to calculate than two parameters distributions (Beta and Kumaraswamy). To achieve fast results properly describing fibre distribution without complicated calculations the two parameters Beta or Kumaraswamy distributions may be applied. Yet the results will not be as precise as for the GT-Kw statistical distribution.

After calculation parameters of probability distributions the results were visualised for the GT-Kw distribution (see Table 5) which best suited to the fibre distribution obtained from the tested specimens. The charts show distribution of fibres along the *Z* axis for sectors 1 to 10 (see Figure 2). Solid lines show experimental results, dotted lines show calculated distribution. One can see strong dependence between values of vibrating time and consistency and the amount of fibres in sectors. The most diverse amount of fibres in various sectors in the cross-sections occurred between vibrating time 240 s and Vebe time 4 s (chart marked red) and 2 s and vibrating time 20 s and Vebe time 2 s (chart marked blue). Vebe time 7 s and 2 s corresponds to normal and liquid consistency of concrete. One can observe superb fit of the statistical distribution GT-Kw (dashed line) to the experimentally tested fibre distribution (solid line, see Table 5). 

One can also observe influence of mould walls on fibre distribution, so-called “wall effect” [43]. The number of fibres in the tenth sector is in any case lower than in the adjacent ninth sector (see Table 5).

Images of points of “piercing” the cross-section plane (see Table 6) by the calculated fibres computed and generated by the CHI-Curvefit program on the basis of GT-Kw distribution show diversity of fibre distribution for different vibrating time and Vebe consistency. There is clear tendency for fibres to settle for mixes characterised by Vebe time 2 s (see Table 6) it is natural for steel fibres which have a relatively high specific gravity to settle in the liquid mixtures. For a Vebe time of 4 s, the fibres tend to settle down during prolonged vibration time (240 s). However, this tendency is no longer noticeable for mixtures characterized by a Vebe time of 7 s. One can see similarity between real fibre distribution in a cross-section of specimens (see Figure 1) and calculated on the basis of GT-Kw distribution (see Table 6, charts marked blue and green) for specified vibrating time and consistency Vebe.

## 5. Conclusions

1. Using the discussed statistical probability distributions it is possible to properly describe fibre distribution considering its random nature and the influence of gravity on fibre settlement.
2. On the basis of chi-square (χ^2^) testing it can be stated that the GT-Kw probability distribution is characterised by the best fitting of the curve and is more suitable for describing fibre distribution of steel fibres in the composite space than other discussed distributions. The model based on GT-Kw distribution can precisely describe fibre distribution.
3. Multi-parameter distributions (such as for example the GT-Kw distribution) are more complex in calculations and require more time to calculate than two parameters distributions (such as Beta and Kumaraswamy distributions), yet bearing in mind using modern computer technology enabling quick calculations and optimised programs the advantage of GT-Kw distribution is essential.
4. To achieve fast results properly describing fibre distribution without complicated calculations the two parameters Beta or Kumaraswamy distributions may be applied. Yet the results will not be as precise as for the GT-Kw statistical distribution.

## Figures and Tables

**Table 1:** The experiment design. Number of specimens in the experiment.

Vibrating Time *t_vib_* (s)	Consistency (Vebe) *t_Vebe_* (s)		
	2	4	7
20	3	3	3
60	3	3	3
240	3	3	3

**Table 2:** Concrete composition.

Ingredient	Amount (kg/m^3^)
Portland cement 42.5R A-V *	420
Sand 0–4 mm	1570
Silica fume	21
Tap water	160–200
Superplasticizer **	16.8
Steel fibre (length 50 mm, diameter 0.8 mm)	117 (*V_f_* = 1.5%) ***

* [40], ** [41], *** *V_f_*-fibre volume fracture.

**Table 3:** The calculated parameters of four statistical distributions ensuring the best fit with experimentally tested fibre distribution along the vertical axis.

Vibrating Time *t_vib_* (s)	Consistency *t_Vebe_* (s)	Beta Distribution F (z; a, b)	Kumaraswamy Distribution F (z; α, β)	TPB Distribution F (z; p, γ, δ)	GT-Kw Distribution F (z; λ, a, b, c, d)
20	7	1.19, 1.24	1.09, 1.13	0.77, 1.25, 1.51	0.30, 0.90, 3.80, 0.90, 0.20
20	4	1.11, 1.15	1.09, 1.14	1.49, 1.15, 0.75	2.97, 0.15, 0.41, 1.12, 1.00
20	2	1.00, 1.16	0.95, 1.10	0.65, 1.17, 1.52	0.38, 0.05, 2.58, 1.28, 0.00
60	7	1.20, 1.37	1.09, 1.27	1.49, 1.36, 0.81	2.70, 0.05, 0.48, 1.29, 0.00
60	4	0.92, 1.14	0.92, 1.14	1.47, 1.13, 0.63	0.31, 0.05, 3.25, 1.19, 1.00
60	2	0.75, 1.60	0.79, 1.64	1.49, 1.57, 0.54	0.19, 0.10, 4.37, 1.78, 0.90
240	7	0.76, 0.99	0.72, 0.97	1.40, 0.99, 0.54	0.09, 0.10, 8.58, 1.14, 0.00
240	4	0.99, 2.16	0.99, 2.14	1.50, 2.11, 0.70	1.10, 1.39, 0.90, 1.10, 0.60
240	2	1.19, 2.55	1.16, 2.64	1.49, 2.51, 0.83	0.61, 2.19, 2.34, 0.14, 0.26

**Table 4:** Chi-square test (χ^2^) results comparison.

Vibrating Time *t_vib_* (s)	Consistency *t_Vebe_* (s)	Beta Distribution	Kumaraswamy Distribution	TPB Distribution	GT-Kw Distribution
20	7	8.74	10.33	8.72	8.43
20	4	13.63	13.66	13.58	13.29
20	2	13.44	13.44	13.13	12.19
60	7	5.52	5.56	5.49	5.35
60	4	10.01	10.01	9.98	9.75
60	2	13.88	13.45	13.09	12.43
240	7	12.68	13.15	12.67	12.33
240	4	13.86	13.84	12.69	8.96
240	2	12.69	12.90	12.60	12.45

**Table 5:** Experimentally tested distribution of fibres (ED*) along the height of the specimens and calculated statistical distribution using the GT-Kw distribution (SD**).

Vibrating Time (s)	Vebe (s)		
	7	4	2
20	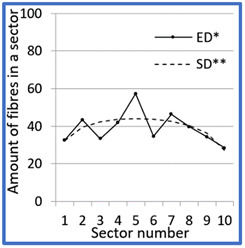	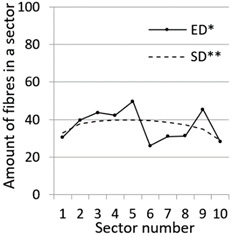	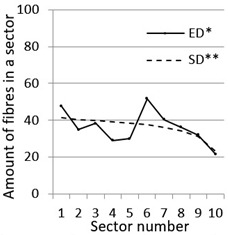
60	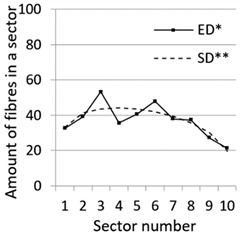	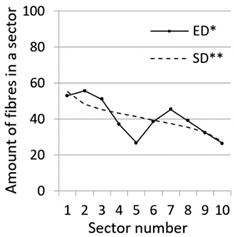	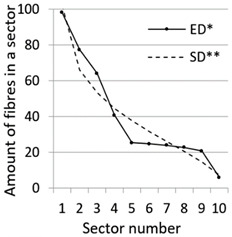
240	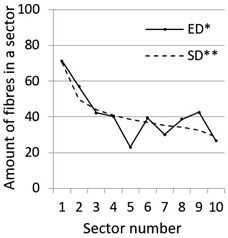	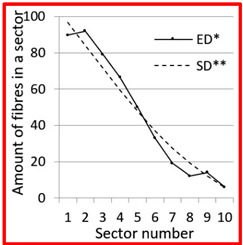	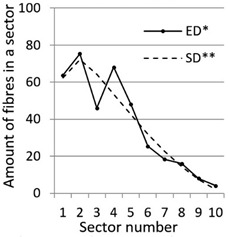

**Table 6:** The calculated fibre distribution in a cross-section of a cubic specimen. Data was generated using GT-Kw probability distribution with parameters corresponding to various vibration time and Vebe consistency (see Table 3 and Table 4).

Vibrating Time (s)	Vebe (s)		
	7	4	2
20	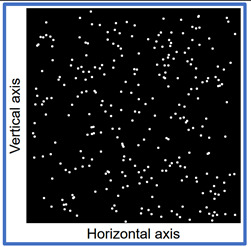	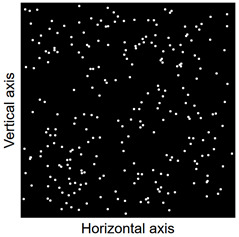	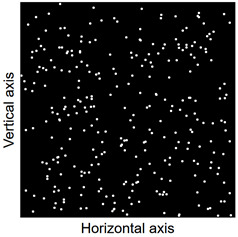
60	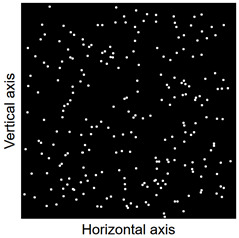	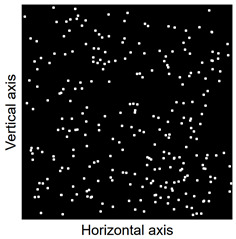	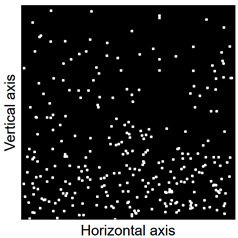
240	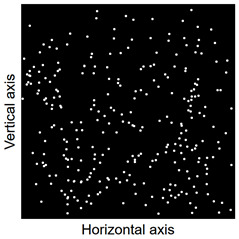	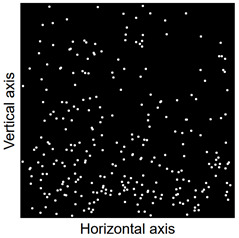	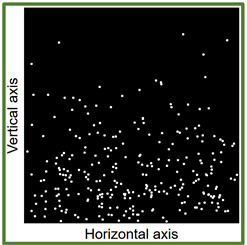

## Data Availability

Not applicable.
